# Evidence of Spillover and Recombination Between Domestic Pigs and Wild Boars Provides New Insights into Porcine Circoviruses

**DOI:** 10.3390/pathogens14121283

**Published:** 2025-12-13

**Authors:** Bernardo Almeida, Margarida D. Duarte, Ana Duarte, Sílvia C. Barros, Fábio Abade dos Santos, Ana Margarida Henriques

**Affiliations:** 1 National Institute of Agrarian and Veterinarian Research, Quinta Do Marquês, Av. da República, 2780-157 Oeiras, Portugal; bsa.almeida2002@gmail.com (B.A.); margarida.duarte@iniav.pt (M.D.D.); ana.duarte@iniav.pt (A.D.); silvia.santosbarros@iniav.pt (S.C.B.); fabio.abade@iniav.pt (F.A.d.S.); 2 Centre for Interdisciplinary Research in Animal Health, Faculdade de Medicina Veterinaria, Universidade de Lisboa, Avenida da Universidade Tecnica, 1300-477 Lisbon, Portugal; 3 Associate Laboratory for Animal and Veterinary Sciences (AL4AnimalS), Avenida da Universidade de Lisboa, 1300-477 Lisbon, Portugal; 4 Departamento de Ciências da Vida, Faculdade de Ciências e Tecnologia, Universidade Nova de Lisboa, Campus da Caparica, 2829-516 Caparica, Portugal; 5 Centro de Ciência Animal e Veterinaria, Faculdade de Medicina Veterinária de Lisboa, Universidade Lusófona, Centro Universitário de Lisboa, 1749-024 Lisbon, Portugal

**Keywords:** One Health, phylogenetic analysis, phylogeography, recombination, transmission pathways

## Abstract

Porcine circovirus types 2 (PCV2) and 3 (PCV3) are major pathogens affecting swine health and productivity, yet important gaps remain in understanding their evolution and circulation in Europe, particularly within wild boar populations that may serve as reservoirs. This study examined the genetic diversity and evolutionary dynamics of PCV2 and PCV3 in Portugal, drawing on viral genomes obtained from domestic pigs and wild boars to explore transmission patterns, spillover events and the contribution of recombination to viral emergence. We identified two PCV2 genotypes (PCV2a and PCV2d) and two PCV3 genotypes (PCV3-2a and PCV3-3g) circulating in Portuguese swine. Phylogeographic reconstruction revealed multiple introductions of both PCV2 and PCV3 from China into Europe, followed by regional diversification and subsequent spread within European wild boar populations. Evidence of bidirectional viral exchange between domestic pigs and wild boars was also observed. Recombination played a notable role in PCV2 evolution, with consistent signals detected among PCV2a sequences and indications that the PCV2h genotype likely originated from a recombinant event involving a Portuguese PCV2a strain and a Chinese PCV2d strain. By contrast, no recombination was detected in PCV3, suggesting that its evolution is primarily mutation-driven. Overall, these findings highlight the complex evolutionary history of swine circoviruses in Europe and underscore the importance of continuous genomic surveillance in both domestic and wild hosts. The study reinforces the value of a One Health approach for monitoring and controlling emerging circoviruses with implications for animal health and livestock production.

## 1. Introduction

Porcine circoviruses are small, non-enveloped, circular single-stranded DNA viruses belonging to *Circoviridae* family and the *Circovirus* genus. Of the four known porcine circovirus species, *Porcine circovirus* 2 (PCV2) and *Porcine circovirus* 3 (PCV3) are the most relevant to swine health. PCV2 is frequently associated with multiple health conditions in pigs, collectively known as porcine circovirus-associated diseases (PCVAD). These include postweaning multisystemic wasting syndrome (PMWS), which is responsible for significant economic losses in the global pig industry [1,2]. PCV3 was first detected in the United States in 2016 [3] and has since been reported worldwide in both healthy and sick pigs. However, its correlation to disease emergence remains unclear [4,5]. PCV2 is currently classified into eight genotypes (PCV2a–h). Before the mid-2000s, PCV2a was the prevalent genotype, then it shifted to PCV2b, and more recently to PCV2d. These genotype changes are the result of recombination events, the immune pressure associated with widespread vaccination, and the global movement of livestock. These factors facilitate the emergence and dissemination of novel viral variants. In contrast, the classification of PCV3 into different genotypes is much less defined. Different research groups have proposed conflicting nomenclature systems based on various criteria, ranging from specific amino acid changes in the capsid protein to full-genome or ORF2 sequence comparisons [6,7,8]. This has led to major inconsistencies in the literature, with several non-equivalent genotype labels in use simultaneously. This hinders broader analyses and a comprehensive understanding of the virus. Although recent efforts by Franzo et al. [9] and Chung et al. [10] have attempted to establish unique PCV3 classification systems, universal consensus has yet to be reached, which continues to hinder comparative analyses and coordinated global surveillance of PCV3.

Wild boars play a key role in maintaining the circulation of PCV2 and PCV3 viruses and facilitating their transmission to domestic pigs. Both viruses have been repeatedly detected in wild boar populations across Europe, including Spain, Italy, Germany and Central Europe [11,12,13,14,15], confirming their role as a reservoir capable of sustaining viral circulation independently of domestic herds. The cross-species spillover events documented across Europe result from overlapping habitats, direct contact, and the movement of animals, including through trade and hunting activities. Such spillovers reduce the effectiveness of vaccine in domestic herds, particularly against PCV2 genotypes not fully covered by current vaccines. Nevertheless, surveillance of wild populations remains limited, resulting in knowledge gaps regarding viral diversity, transmission dynamics, and the potential role of wildlife in shaping viral evolution. To better understand the epidemiological role of wild boars in PCV2 and PCV3 circulation, further steps are required, including expanded longitudinal sampling, integrated wildlife–livestock surveillance programs, and genomic monitoring to detect genotype shifts and recombination events. Closing these knowledge gaps is essential for developing effective control strategies and implementing coordinated, cross-border monitoring programs.

Against this backdrop, the present study examines the circulation, genetic diversity and evolutionary relationships of PCV2 and PCV3 in both domestic pigs and wild boars, employing a combination of phylogenetic, phylogeographic and recombination analyses. This multifaceted approach provides a clearer picture of the relationship between Portuguese strains and international viral lineages, particularly in Europe and Asia. This highlights the importance of ongoing surveillance in line with a One Health approach.

## 2. Materials and Methods

### 2.1. Sample Selection

The samples included in this study were collected and characterized in a previously published study that detailed their origin and the diagnostic methods for PCV2 and PCV3 detection [16]. From that dataset, four PCV2-positive samples (three from domestic pigs and one from a wild boar) and two PCV3-positive samples (one from a domestic pig and one from a wild boar, previously subjected to partial sequencing, were randomly selected for whole-genome sequencing. The corresponding GenBank accession numbers are PV753264–PV753267 for PCV2 and PV753268 and OR479851 for PCV3 samples.

### 2.2. Amplification of PCV2-Positive Samples by PCR and Sanger Sequencing

The complete PCV2 genome of the selected samples was sequenced by the Sanger method, after PCR amplification using different primer combinations able to amplify overlapping regions of the genome (Table 1). Amplification was performed using the NZYTaq II 2x Green Master Mix (NZYTech, Lisbon, Portugal), with 1 µM of each primer, in a total volume of 25 µL. The amplification program consisted of an initial denaturation step at 95 °C for 2 min, followed by 40 cycles of denaturation at 95 °C for 30 s, annealing at 58 °C for 30 s and extension at 72 °C for 90 s, and a final extension step at 72 °C for 5 min.

Amplification products were observed on a 1% agarose gel, excised and purified with the NZYGelpure Kit (NZYTech, Lisbon, Portugal).

In the Sanger sequencing each reaction included 1 µL of 5× sequencing buffer and 2 µL of sequencing mix from the BigDye™ Terminator v3.1 Cycle Sequencing Kit (Thermo Fisher Scientific, Waltham, MA, USA), 0.5 µM primer and an appropriate volume of DNA, adjusted based on fragment concentration. Nuclease-free water was added to a 10 µL total volume. The sequencing protocol consisted of an initial denaturation at 96 °C for 1 min, followed by 25 cycles of denaturation at 96 °C for 10 s and annealing/extension at 60 °C for 70 s. Sanger sequencing products were then cleaned and concentrated using the Zymo Research ZR DNA Sequencing Clean-up Kit™ (Zymo Research, Irvine, CA, USA) following the manufacturer’s protocol. Fragments were separated using a 3130 Genetic Analyzer (Thermo Fisher Scientific, Waltham, MA, USA) and the sequence reads were assembled with SeqScape v2.5 software (Thermo Fisher Scientific, Waltham, MA, USA).

### 2.3. Amplification of PCV3-Positive Samples by Sequence-Independent Single Primer Amplification (SISPA)

The full-genome amplification of the selected PCV3 samples was performed with the Sequence-Independent Single Primer Amplification (SISPA) protocol.

First-strand cDNA was generated using the SuperScript™ IV First-Strand Synthesis System (Thermo Fisher Scientific, Waltham, MA, USA). The reaction mixture, containing 5 μL of primer FR20-N (10 pmol/μL) (Table 1), 1 μL of 10 mM dNTP mix, 5 μL of DNA template and 2 μL of nuclease-free water, was incubated at 65 °C for 5 min and cooled on ice for 5 min. Subsequently, 4 μL of 5× SuperScript IV Reaction Buffer, 1 μL of 100 mM DTT, 1 μL of Ribonuclease Inhibitor and 1 μL of SuperScript IV Reverse Transcriptase were added. Reverse transcription was carried out under the following conditions: 23 °C for 10 min, 50 °C for 45 min and 80 °C for 10 min followed by cooling on ice. Then, 1 μL of RNase H was added and the mixture was incubated at 37 °C for 20 min.

Double-Stranded cDNA (ds-cDNA) was generated using the DNA Polymerase I, Large (Klenow) Fragment kit (New England Biolabs, Ipswich, MA, USA). The reaction mix contained 2.5 μL of 10× NEBuffer, 0.5 μL of 10 mM dNTPs, 20 μL of first-strand cDNA from the previous reaction and 1 μL of the Klenow fragment enzyme. The mixture was incubated at 25 °C for 15 min. The reaction was terminated by adding 0.5 μL of 10 mM EDTA, followed by heat inactivation at 75 °C for 20 min.

Double-stranded cDNA was amplified by PCR in a 50 μL reaction containing 5 μL of ds-cDNA, 25 μL of 2× Speedy Master Mix (SPEEDY NZYTaq 2× Colourless Master Mix from NZYTech, Lisbon, Portugal), 5 μL of primer FR20 (10 pmol/μL) (Table 1) and 15 μL of nuclease-free water. PCR amplification was carried out with an initial denaturation at 95 °C for 2 min, followed by 30 cycles of 95 °C for 30 s, 55 °C for 20 s and 72 °C for 2 min, ending with a final extension at 72 °C for 10 min.

PCR products were analyzed on a 1% agarose gel stained with GreenSafe (NZYTech, Lisbon, Portugal) and observed using a GelDoc Go Imaging System (Bio-Rad, Hercules, CA, USA).

### 2.4. MinION Sequencing of PCV3-Positive Samples

The PCV3-positive samples sequencing was conducted using MinION platform (Oxford Nanopore Technologies) with the Rapid Barcoding Sequencing Kit (SQK-RBK114.24, protocol RBK_9176_v114_revM_27Nov2022), according to the manufacturer’s instructions. In the library preparation step, 200 ng of purified genomic DNA per sample were used. Flow cell priming and loading was performed in a MinION Flow Cell R10.4.01 (FLO-MIN114). Sequencing was conducted on a MinION Mk1B device for 3 h, using MinKNOW v24.11.10 with a high-accuracy base-calling model (minimum Q-score = 9).

The multiple files generated during sequencing and base-calling were merged into a single file and analyzed with a custom Python script that also facilitates BAM file inspection for base-call verification and read confidence assessment, available at https://github.com/AlmeidaBernardo/MinION-analysis-scripts (accessed on 3 November 2025).

Merged files were analyzed using the INSaFLU [17,18] and Genome Detective [19] online tools.

### 2.5. Phylogenetic and Phylogeographic Analysis

The sequences were selected assuring geographical and temporal representativeness and the alignment was performed in AliView v1.28 [20] applying the Muscle algorithm. TREE-PUZZLE v5.3 [21,22] was used in the phylogenetic analysis to assess the phylogenetic signal ensuring that the data were appropriate for phylogenetic analysis.

From the complete PCV3 alignment, due to the low phylogenetic signal of the complete genome dataset, 15 derivative datasets were constructed, each representing a unique combination of coding regions with the stop codons removed. These datasets encompassed every possible arrangement of the three major open reading frames, following the methodology of Chung et al. [10].

The phylogenetic analysis was performed via command-line using IQ-TREE v3.0.1 [23,24,25]. The ModelFinder Plus option (-m MFP) was employed to identify the best-fit substitution model for the datasets and subsequently infer the maximum likelihood. Branch support was evaluated using ultrafast bootstrap approximation and SH-aLRT tests, each with 10,000 replicates (-bb 10,000 -bnni -alrt 10,000), ensuring robust statistical confidence in the inferred clades.

The maximum likelihood trees were visualized and edited in FigTree v1.4.4, displaying bootstrap support values above 70%, and the temporal signal of each dataset was assessed through root-to-tip regression analysis in TempEst v1.5.3 [26] using the phylogenetic tree inferred with IQ-TREE to confirm if the dates inferred in the phylogeographic analysis were reliable.

The same sequence dataset used for the phylogenetic analysis was employed for the phylogeographic analysis, which was conducted using the Nextstrain Augur pipeline [27], within the INSaFLU platform. The resulting phylogeographic reconstruction was visualized using Auspice (https://auspice.us accessed on 3 November 2025).

### 2.6. Recombination Analysis

The RDP4 v.4.101 software was used to perform recombination analysis [28], employing the following statistical methods to detect recombination signals: RDP [29], GENECONV [30], Bootscan [28], MaxChi [31], Chimaera [32], SiScan [33], 3Seq [34], BURT [28], PhylPro [35], VisRD Occupancy [36], TOPAL DSS [37] and LARD [38]. The recombination events considered robust were those detected by a minimum of three independent methods and statistically supported with *p*-values below 5 × 10^−2^.

## 3. Results

**Figure 1:**
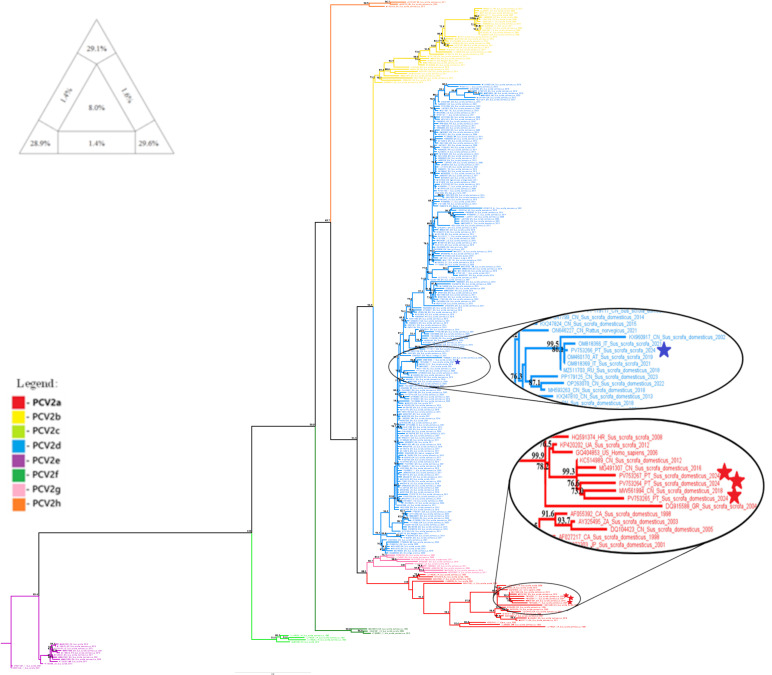
Evolutionary analysis of PCV2 inferred in IQ-TREE by using the Maximum Likelihood method and the best-fit model GTR + F + R4 model (General Time Reversible model). The tree with the highest log likelihood (−15,347.224) is shown. The phylogenetic analysis was performed using the full PCV2 genome of 308 sequences, with model selection performed by ModelFinder Plus and branch support assessed with 2000 ultrafast bootstrap replicates and 2000 SH-aLRT replicates. Sequences marked with a star represent samples obtained in the present study. Only the bootstrap values higher than 70% are shown in the tree. The triangular diagram on the left side of the phylogenetic tree represents the quartet puzzling support topology obtained with TREE-PUZZLE for this dataset. The percentage of resolved quartets is 87.6% (corresponding to the sum of the three values at the triangle’s tips).

The PCV2 dataset, comprising 308 whole-genome sequences, exhibited high phylogenetic resolution in the TREE-PUZZLE analysis, with 87.6% of quartets being resolved (see triangular diagram in Figure 1). The PCV2 phylogenetic analysis was then performed and confirmed the classification of the domestic pig samples PV753264, PV753265 and PV753267 into the PCV2a clade, and of the wild boar sample PV753266 into the PCV2d clade. These are the same clades that were identified in our previous study, in which phylogeny was conducted using only partial genome sequences [16].

**Figure 2:**
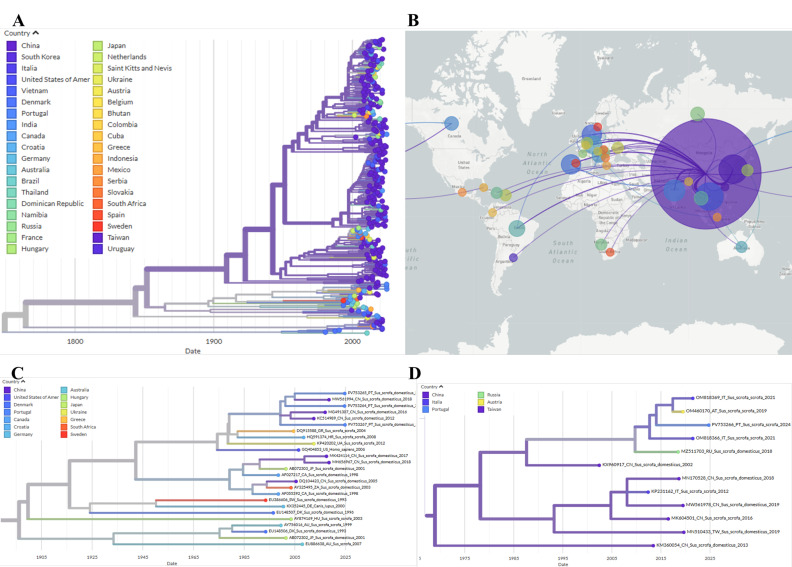
Auspice view of the PCV2 tree generated using the INSaFLU/Nextstrain pipeline and corresponding world map of the estimated transmission pathways. (**A**)—Phylogenetic tree used for phylogeographic analysis. Colors represent country of origin and branch length represent time; (**B**)—Map showing transmission lines portraying potential viral dispersion routes; (**C**)—Expanded view of the tree branch containing sequences PV753264, PV753265 and PV753267; (**D**)—Expanded view of the tree branch containing sequence PV753266.

Phylogeographic reconstruction indicates that the PV753266 sequence emerged in China and was introduced to Portugal, subsequently spreading to wild boar populations in Italy and Austria. Sequences PV753264, PV753265, and PV753267 form a distinct lineage that also originates from China, and later dispersing across Europe and being detected in wild boars in Portugal, Croatia, Ukraine and Greece. The lineage was then reintroduced to China from Portugal (Figure 2).

The PCV2 phylogenetic analysis (Figure 1) shows that sequences PV753264, PV753265 and PV753267 are highly similar. Thus, recombination events identified in one sequence were also detected in the others. RDP4 analysis identified two distinct recombination events involving this group of sequences. No recombination events were detected in sequence PV753266.

**Figure 3:**
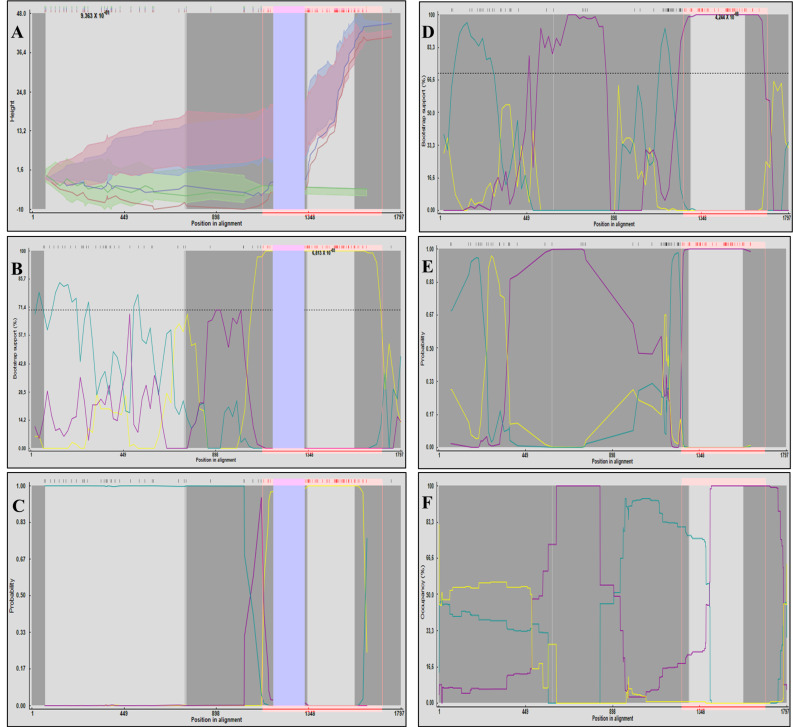
Graphical outputs of recombination analyses performed in RDP4 concerning PCV2 sequences. (**A**) 3Seq, (**B**) Bootscan, and (**C**) BURT graphics illustrating the recombination event that originated the sequence PV753265. In the 3Seq graphic, the red line represents the Recombinant (PV753265), the green line the Major Parent (KX960917), and the blue line the Minor Parent (KT819170). In the Bootscan and BURT plots, the yellow line represents similarity between the Major and Minor Parents, the blue line between the Recombinant and the Major Parent, and the purple line between the Recombinant and the Minor Parent. The blue-shaded region (positions 1170–1325 nt) indicates the 155 nt segment excluded from analysis by all three methods. (**D**) Bootscan, (**E**) BURT, and (**F**) VisRD Occupancy graphics illustrating the recombinant event that originated the Recombinant sequence (MH465453). In the three graphics, the yellow line represents similarity between the Major (PV753265) and Minor Parents (KC515014), the blue line between the Recombinant and the Major Parent, and the purple line between the Recombinant and the Minor Parent.

The first recombination event was supported by the 3Seq, BURT, Bootscan, MaxChi, SiScan, and LARD methods (all *p* < 2.60 × 10^−2^), indicating that the sequences likely resulted from a recombination event between the sequences KX960917 (the major parent) and KT819170 (the minor parent). Both sequences were identified as PCV2d sequences in the phylogenetic analysis. A 155-nt region (positions 1170–1325), which includes an 11-nt insertion present in 12 sequences, was automatically excluded by some detection methods. Similar results were obtained when the analysis was repeated without these sequences, with recombination again being detected by multiple methods: GENECONV, Bootscan, SiScan, 3Seq, BURT, and VisRD Occupancy (all *p* < 2.45 × 10^−2^). Breakpoints were consistently located between 830 and 1170 nt, across all methods. The PV753265 sequence showed greater similarity to the major parent in the 0–830 nt region and to the minor parent in the 1170–1797 nt region (Figure 3A–C).

The second event involved the parental sequences PV753265 (PCV2a) and KC515014 (PCV2d), which contributed to the emergence of the PCV2h genotype in China. This event was supported by the Bootscan, MaxChi, SiScan, BURT and VisRD Occupancy methods (all *p* < 1.33 × 10^−2^), as shown in Figure 3D–F. These analyses identified consistent breakpoint regions around nucleotides (nt) 350–420, 890–900 and 1180–1250.

The dataset containing the PCV3 coding regions in the order Rep + ORF3 + Cap exhibited the highest phylogenetic resolution among the 16 datasets, with 84.3% of resolved quartets in the analysis conducted with TREE-PUZZLE. Therefore, this dataset was used for phylogenetic analysis. Individual TREE-PUZZLE analyses for the 15 datasets are included in Supplementary Figure S1.

**Figure 4:**
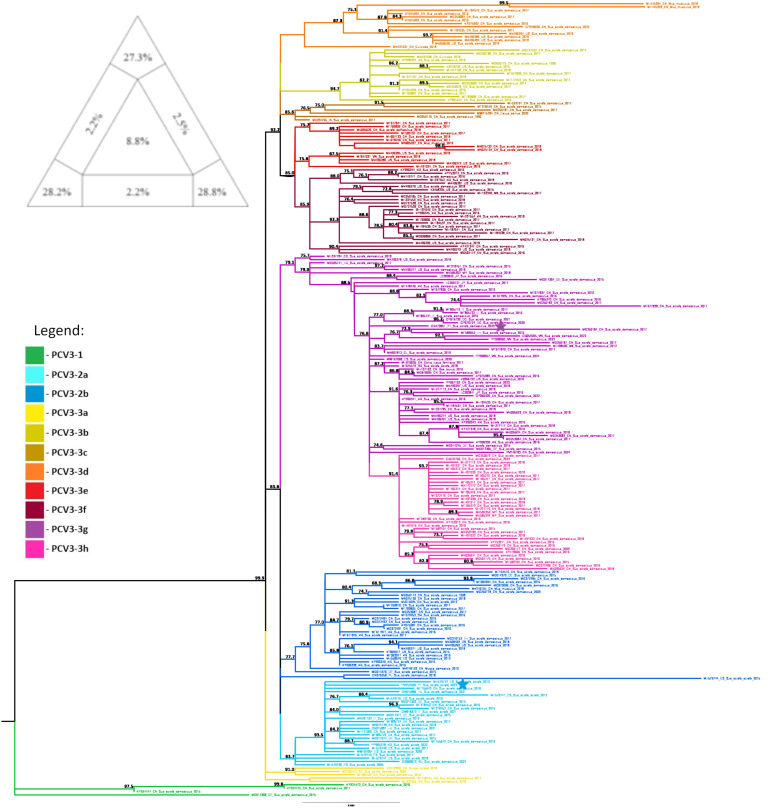
Evolutionary analysis of PCV3 inferred in IQ-TREE by using the Maximum Likelihood method and TIM + F + R3 model (Transitional model). The tree with the highest log likelihood (–11387.853) is shown. The phylogenetic analysis was performed using the coding regions of the PCV3 genome (in the order Rep + ORF3 + Cap) of 239 sequences, with model selection performed by ModelFinder Plus and branch support assessed with 10,000 ultrafast bootstrap replicates (with NNI correction) and 10,000 SH-aLRT replicates. Sequences marked with a star represent samples obtained in the present study. Only the bootstrap values higher than 70% are shown in the tree. The triangular diagram on the left side of the phylogenetic tree represents the quartet puzzling support topology obtained with TREE-PUZZLE for this dataset (Rep + ORF3 + Cap dataset). The percentage of resolved quartets is 84.3% (corresponding to the sum of the three values at the triangle’s tips).

The PCV3 sequences obtained in this study clustered into two well-supported clades: PCV3-2 subtype a (wild boar sample PV753268) and PCV3-3 subtype g (domestic pig sample OR479851), as illustrated in Figure 4.

**Figure 5:**
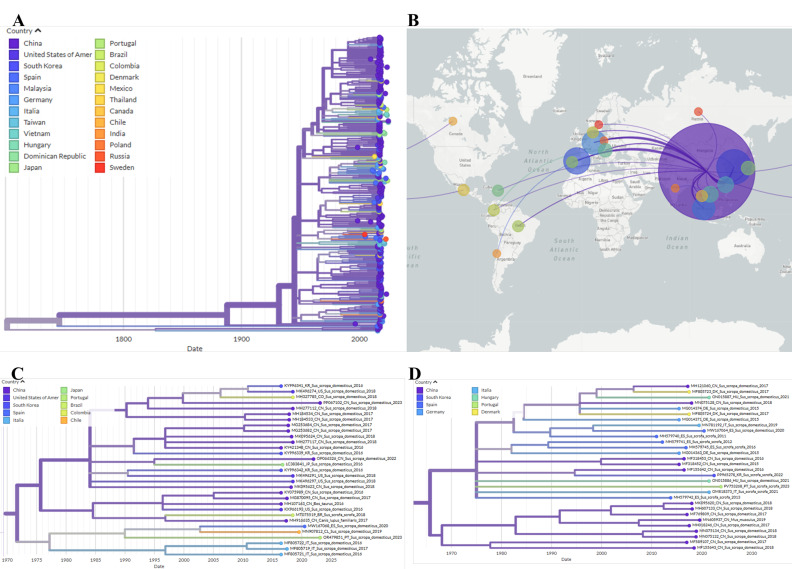
Auspice view of the PCV3 tree generated using the INSaFLU/Nextstrain pipeline and corresponding world map of the estimated transmission pathways. (**A**)—Phylogenetic tree used for phylogeographic analysis. Colors represent country of origin and branch length represent time; (**B**)—Map showing transmission lines portraying potential viral dispersion routes; (**C**)—Expanded view of the tree branch containing sequence OR479851; (**D**)—Expanded view of the tree branch containing sequence PV753268.

Phylogeographic analysis suggested that strain OR479851 originated in China and spread to Spain via Portugal, whereas sequence PV753268 likely represents a strain that circulates among European wild boars (Figure 5). No recombination was detected for either sequence in RDP4.

**Figure 6:**
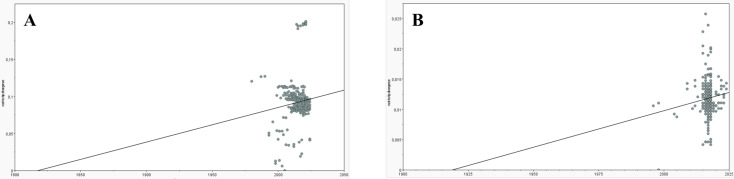
Root-to-tip regression analysis performed with TempEst. Gray dots represent sequences, plotted according to its genetic divergence from the inferred root of the tree (y-axis, substitutions per site) against its sampling date (x-axis, in years). The black line represents the best-fit regression of root-to-tip distances over time. In the PCV2 graphic (**A**) the line’s slope is 4.67 × 10^−4^ substitutions/site/year, the correlation coefficient is 0.11 and the coefficient of determination R^2^ equals 0.012. In the PCV3 graphic (**B**) the line’s slope is 1.21 × 10^−4^ substitutions/site/year, the correlation coefficient is 0.13 and the coefficient of determination R^2^ equals 0.016.

TempEst analysis revealed positive slopes (4.67 × 10^−4^ substitutions/site/year for PCV2 and 1.21 × 10^−4^ for PCV3), but these were associated with extremely low correlations (R^2^ = 0.012 and R^2^ = 0.016, for PCV2 and PCV3, respectively), indicating an absence of strong clock-like structure (Figure 6). While TempEst estimates the time to the most recent common ancestor (tMRCA) at 1818 for PCV2 and 1919 for PCV3, the phylogeographic reconstruction estimated much older dates (1789 for PCV2 and 1701 for PCV3, respectively), but with no confidence in the estimates. These results reinforce the absence of a strong temporal signal in either dataset, which prevents dependable molecular clock calibration and accurate divergence dating.

## 4. Discussion

This study provides new evidence of cross-species transmission between wild and domestic swine populations, as well as recombination events involving Portuguese PCV2 strains, both of which appear to play a central role in shaping viral evolution and diversity. The detection of PCV2d in wild boars represents a significant threat to the Portuguese swine industry due to the risk of spillover to domestic pig populations, as most vaccines primarily target PCV2a, with only a few also covering PCV2b [39]. This risk is particularly high in extensive production systems, where biosecurity measures are limited and contact between the two subspecies is difficult to prevent.

PCV2d is more virulent than PCV2a and PCV2b [40,41] and has been linked to reduced vaccine efficacy [42]. However, vaccines formulated against PCV2b remain a practical option, as they provide cross-protection against the PCV2d genotype [39].

Phylogenetic analysis revealed that the PCV2d sequence PV753266 belongs to a group of wild boar sequences from Italy and Austria (2019–2021). This group, in turn, clustered with domestic pig sequences from China (2002) and Russia (2018). Phylogeographic analysis suggests that this strain was introduced to Europe via China, around 2009 (confidence interval (CI) = 1451–2013) and its spread from Portugal to Italy in 2017 (CI = 1845–2018), from where it has reached Austria. These results imply that this strain was introduced to Europe via domestic pigs from Asia and subsequently spilled over into the wild boar population, where it now circulates endemically.

The PCV2a sequences PV753264, PV753265, and PV753267 clustered with Chinese domestic pig sequences from 2012 to 2018, which in turn, grouped with older wild boars isolates from in Croatia, Ukraine and Greece. Phylogeographic analysis indicated that this PCV2a strain likely originated in China, entering Europe on at least three occasions. It eventually reached Portugal in 1999 (CI = 822–2012), Ukraine, and Croatia. From there, it spread to Greece and from Portugal back to China in 2004 (CI = 979–2018).

Recombination signals in PV753265 were consistent across methods, although the location of the breakpoints was limited by sequence similarity and the potential occurrence of multiple recombination events.

Despite the difficulty in locating the exact breakpoints, clear patterns were observed. Sequences PV753264, PV753265 and PV753267 shared the same recombination signature as six other sequences, with all clustering within a strongly supported PCV2a branch (bootstrap = 99.9). This suggests that the recombination between two PCV2d viruses may have given rise to the PCV2a clade. A comparable pattern was also observed for the PCV2h clade, which appears to have resulted from a recombination event between the Portuguese PCV2a sequence PV753265 and the Chinese PCV2d sequence KC515014. This signal was consistently detected in all three sequences forming the PCV2h cluster (bootstrap = 99.5). These results suggest that not only did PV753265 originate through recombination, it also contributed as a parental sequence to the emergence of a distinct genotype. This reinforces the idea that recombination is a major driver of PCV2 diversification [43,44,45].

The detection of a recombination event between two PCV2d strains yielding a sequence classified as PCV2a, while initially paradoxical, can be explained by the high frequency of recombination in PCV2 viruses and the mosaic nature of circulating strains. This event can be explained by a model that supposes that parental strains are not ‘pure’ PCV2d genotypes but are themselves mosaics. In a viral population characterized by extensive recombination (observed in 77% of the 308 PCV2d strains analyzed in this study), many circulating PCV2d strains have likely acquired genomic fragments from other genotypes through prior recombination events. These mosaic strains may still be classified as PCV2d if the majority of their genomes are similar to PCV2d strains. A recombination between two PCV2d mosaic strains may therefore originate a strain that is mostly similar to PCV2a strains and, therefore, is framed in a different genotype. This model explains how two apparently similar parental strains, recombining with each other, may yield a completely different strain.

The transmission event of a PCV2a sequence from Portugal to China, as revealed by the phylogeographical analysis, is consistent with the recombination findings which indicate that the PCV2h genotype, comprising solely of Asian sequences (Chinese and Vietnamese), emerged from a recombination event involving a Portuguese and a Chinese sequence. These findings emphasize the combined impact of recombination and cross-border viral transmission on worldwide genetic diversification of PCV2 [46,47].

The PCV3-2a clade, which the wild boar sequence PV753268 appears to belong to, seems to have originated in Spanish wild boars, as indicated by the three Spanish wild boar sequences at the root of this clade. Further phylogeographic analysis indicates that this lineage likely spread from China to Europe through the introduction of domestic pig strains on multiple occasions between 1975 (CI = 1839–2008) and 2006 (CI = 1912–2017), and is currently circulating among wild boars in several European countries, including Portugal, Italy, and Spain.

The PCV3-3g sequence OR479851 did not cluster with any defined group in the phylogenetic analysis. However, phylogeographic reconstruction suggests an origin in China, from where the strain was likely introduced to Portugal around 1989 (CI = 1858–2019), and subsequently to Spain around 2002 (CI = 1897–2019) and then to Chile.

No recombination events were identified in the analyzed PCV3 sequences (PV753268 and OR479851), which is consistent with previous findings reporting a lack of recombination within PCV3 [48,49]. Unlike PCV2 and other CRESS DNA viruses, for which recombination is a major evolutionary force, PCV3 appears to evolve through different mechanisms. This likely reflects either its more recent emergence or the overall limited genetic variation observed across known PCV3 genomes [50].

Wild boars serve as important reservoirs for PCV2 and PCV3, and their growing populations in Portugal increase the likelihood of contact with domestic pigs, particularly in extensive systems with limited biosecurity. Integrating wild boars into One Health surveillance is therefore essential to monitor viral circulation and detect cross-species transmission. Strengthening basic biosecurity measures, such as effective fencing, controlled access to feed and water, and proper carcass management, can help reduce spillover at the wildlife–livestock interface. Overall, the ecological expansion of wild boars reinforces the need for coordinated surveillance and improved biosecurity to limit the spread of circoviruses into domestic herds.

## 5. Conclusions

Root-to-tip analyses in TempEst revealed a very weak temporal signal for both PCV2 and PCV3 datasets, indicating that the estimated dates are unreliable and explaining the unrealistically wide confidence intervals obtained. While precise dating of transmission events could not be established, meaningful phylogeographic patterns were observed.

The results obtained highlight numerous spillover events between both domestic pig and wild boar populations, emphasizing the need to extend surveillance efforts to wild populations rather than focusing exclusively on domestic herds. Furthermore, the findings clearly reveal transmission routes within Europe and from Asia, particularly China, to Europe. These patterns are consistently observed for both PCV2 and PCV3, reflecting the similar transmission dynamics for the two viruses. The interpretation of the inferred transmission routes should be approached with caution, as these estimates are inherently limited by the availability and geographic distribution of publicly accessible sequences. The absence of strains from neighboring countries limits the ability to detect potential cross-border transmission. Nonetheless, the routes identified represent the most plausible reconstruction supported by the currently available data.

This study integrates phylogenetic, phylogeographic, and recombination analyses, providing new insights into the circulation and evolution of PCV2 and PCV3 in Portugal and Europe more broadly. This multidisciplinary approach has enabled a more comprehensive understanding of the genetic diversity, evolutionary relationships, and transmission dynamics of these viruses, thereby reinforcing the importance of having continued cross-border surveillance programs to monitor these viruses within Europe.

## Figures and Tables

**Table 1:** Primers sequences used for amplifying the PCV2 genome by conventional PCR and PCV3 by SISPA.

Reaction	Primer Name	Sequence	nt Position	Target Gene
PCV2 PCR	CircSacF	5′-CCGCGGGCTGGCTGAACTTTTGAAAGT-3′	491–517	Replicase
	CircEcoF	5′-GAATTCAACCTTAACCTTTCTTATTCT-3′	1420–1446	Capsid
	PCV2-SeqF	5′-GCTGCCACATCGAGAAAGCS-3′	289–308	Replicase
	CircSacR	5′-CCGCGGAAATTTCTGACAAACGTTACA-3′	496–470	Replicase
	CircEcoR	5′-GAATTCTGGCCCTGCTCCCCCATCAC-3′	1425–1400	Capsid
	PCV2-SeqR	5′-CACAGTCTCAGTAGATCATCCC-3′	739–718	Replicase
	AS4	5′-CCGCACCTTCGGATATACTGTC-3′	1585–1564	Capsid
PCV3 SISPA	FR20-N	5′-GCCGAAGCTCTGCAGATATCNNNNNN-3′	Random sequences	Random sequences
	FR20	5′-GCCGAAGCTCTGCAGATATC-3′		

## Data Availability

The data supporting the results of this study can be obtained by contacting the corresponding author; however, the right to privacy of the property owners will be respected.

## Supplementary Materials

The following supporting information can be downloaded at: https://www.mdpi.com/article/10.3390/pathogens14121283/s1, Figure S1: Likelihood mapping of 15 PCV3 datasets corresponding to the complete genome dataset (O) and all the possible combinations of the coding regions (Rep, ORF3 and Cap), apart from the Rep + ORF3 + Cap dataset that is already presented alongside the PCV3 phylogenetic tree in Figure 4 (A-N). Each triangular diagram represents the quartet puzzling support topology obtained in TREE-PUZZLE for one of the PCV3 datasets. The values at the triangle tips indicate the percentage of resolved quartets and their sum reflects the total proportion of resolved quartets for that dataset.

## Abbreviations

The following abbreviations are used in this manuscript:

°C	Degrees Celsius
µL	Microliter
µM	Micromolar
BAM	Binary Alignment Map
bp	Base pairs
cDNA	Complementary DNA
CI	Confidence Interval
CRESS	Circular Rep-encoding Single-Stranded
DNA	Deoxyribonucleic acid
dNTP	Deoxyribonucleotide triphosphate
ds-cDNA	double-stranded complementary DNA
EDTA	Ethylenediaminetetraacetic acid
GTR	General Time Reversible
MFP	ModelFinder Plus
ng	Nanogram
nt	Nucleotide
ORF	Open Reading Frame
PCR	Polymerase chain reaction
PCV2	*Porcine circovirus type 2*
PCV3	*Porcine circovirus type 3*
PCVAD	Porcine-circovirus-associated disease
PMWS	Postweaning multisystemic wasting syndrome
RDP4	Recombination Detection Program version 4
SH-aLRT	Shimodaira–Hasegawa-like approximate Likelihood-Ratio Test
SISPA	Sequence-Independent Single Primer Amplification
TIM	Transitional model
× *g*	Relative centrifugal force (RCF)
