# Supplementary figures and images for: Evidence of Spillover and Recombination Between Domestic Pigs and Wild Boars Provides New Insights into Porcine Circoviruses
Source: Pathogens. 2025 Dec 13;14(12):1283. doi: 10.3390/pathogens14121283 (PMC12735692; doi:10.3390/pathogens14121283)

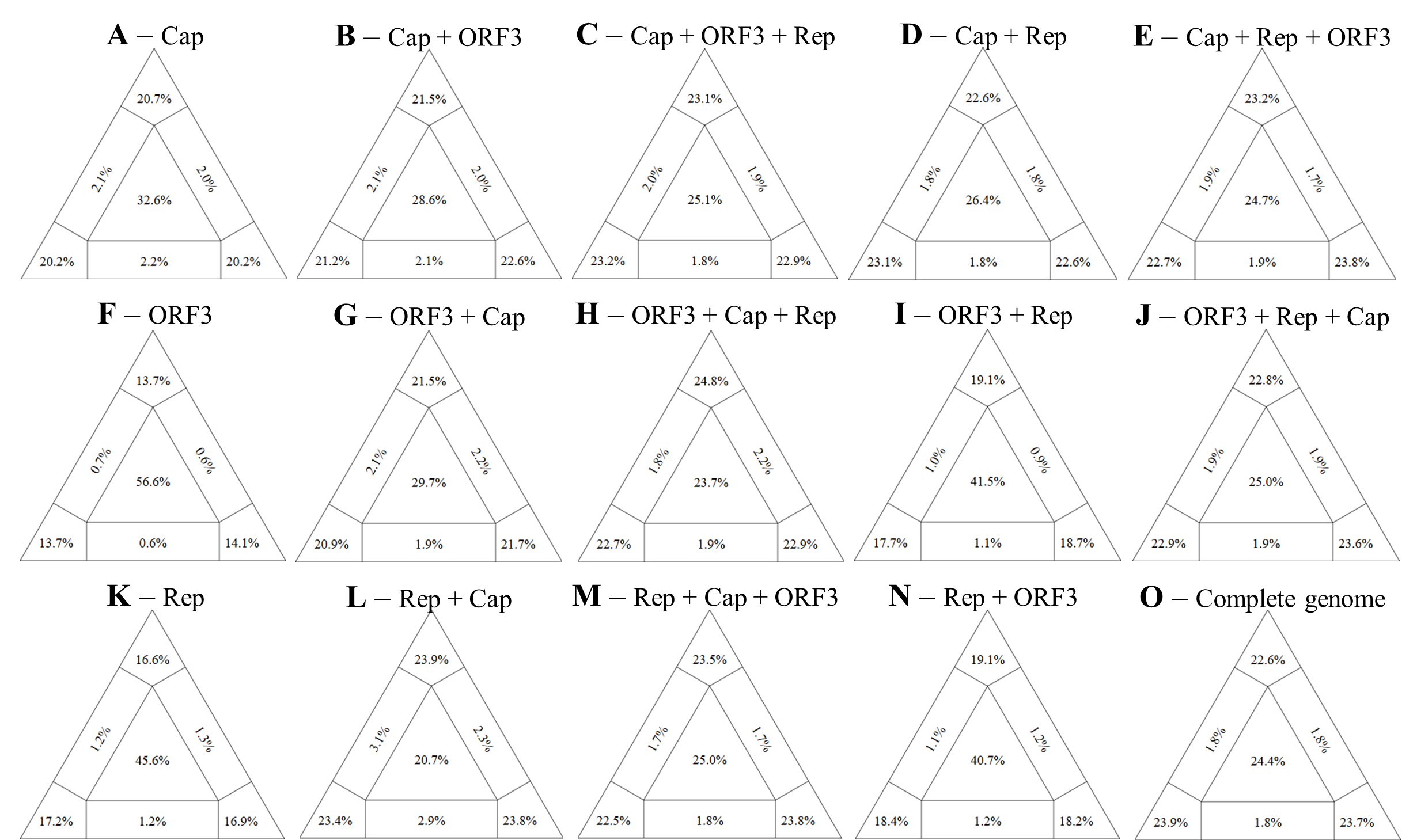

Supplement: Supplementary file 1 [file pathogens-14-01283-s001.zip › pathogens-4004402-Figure S1.png]
